# In memory of Prof. Helena Rašková, MD., DSc., Dr.h.c.

**DOI:** 10.2478/v10102-010-0009-z

**Published:** 2010-06

**Authors:** Viktor Bauer

**Affiliations:** Institute of Experimental Pharmacology & Toxicology, Slovak Academy of Sciences, SK-84104 Bratislava, Slovakia

*“Old-timers, like me (born 1913) fortunately often do not lose the long-term memory of past events. I witnessed how world pharmacology stepped out of shadow of physiology into a generally recognized branch of science, and its international organization International Union of Pharmacology (IUPHAR) became an established member of the International Committee of Scientific Union (ICSU)”.*

*My friends abroad and my pupils at home helped me very much to overcome the difficulties of the twenty years prior the ‘velvet revolution’ In Prague”.*

H. Rašková (1997). Pharmnacol Toxicol 80: 255–261.

On April 13, 2010, **Prof. Helena Rašková**, MD, DrSc., Dr.h.c., the legendary figure of Czecho-Slovak and world pharmacology, passed away. She was born in 1913 in Laussane in a physician's family of a Czech father and Russian mother. She was predestinated for science even due to her childhood experience and meetings with A. Einstein and the later Nobel prize winner W. Hess, who played in a chamber quartet with her father. Moreover, she became a polyglot from the earliest time of her life (Czech from her father, Russian from her mother), Switzerdeutsch and Hochdeutsch from nursary rhymes, French in the elementary school in Zurich and basic English in convent summer school in Weymouth, where as she put it “she got an early lesson that one loves one's own country but is also a citizen of the world”.

Prof. Rašková has significantly contributed to the establishment of pharmacology as an independent scientific discipline in the world context and established the Czecho-Slovak Pharmacological School of the 1950s and 1960s.

She graduated from the Faculty of Medicine of Charles University in Prague in 1937. In 1938 she married K. Raška, a later worldwide known professor of epidemiology.

Her first career in the Medical Department of Charles University was interrupted soon by the 2^nd^ World War and the closure of the Czech universities and institutions of higher education in 1939. During the 2^nd^ World War she accepted the suitable opportunity to work part time as a “factory doctor” for a small pharmaceutical company in Prague (after 1946 nationalized as Spofa Fragner, later on as Synpharma, after privatization in 1998 as Léčiva CZ and from 2004 as part of Zentiva, j.s.c.). The owner Benjamín Fragner employed number of students, microbiologists, chemists who lost their positions when the Universities were closed. Thus she had an opportunity to create contacts with the sciences connected with the development of new medications. Here she learned methods of pharmacology and toxicology from the bottom up. This work motivated her for later lifetime love, pharmacology. After the end of the World War II she exerted a high activity in organizing the background for her husband in the elimination of epidemic typhoid fever in the liberated concentration camp of Terezine.

Soon after the re-opening of the Czech universities in 1945, she became one of the restorers of the Pharmacological Institute of the Faculty of Medicine of Charles University in the well-known historical buildings of Prague, Albertov. She accurately anticipated the trends in pharmacology and became recognized as a leading authority (called at that time and by all of us also later, as “Lady Boss“) among her university collaborators and also in pharmacological research in the gradually renewed pharmaceutical industry. As early as 1946, she established contacts with F. Švec, professor of the Department of Pharmacology, Medical Faculty of the Comenius University in Bratislava.

In 1947 she spent some time at the Department of Pharmacology of the University at Oxford, chaired by J.H. Burn and in 1848 she got a fellowship in Harvard. She owed a lot to this stay and the fantastic spirit of the department for her development in pharmacology and management of science. All foreign students were accepted regardless their country of origin, religion or other characteristics. As she remembered: “*The typical tea time from 11 a.m. to 4 p.m. was always kept. The Department was open 24 hours a day, the University Library until 2 a.m. The only less interesting event for a central European stomach like mine was the lunch at 1 p.m. In general, the conversation was about the description of a good experiment, why and what, not about the last football game or other sports events. The working hours were not fixed but, from time to time, each of us had meetings with the Chairman and experiments were analyzed in detail together with the plans for the next steps.”* The origin of her worldwide scientific contact in pharmacology started in Burn's Department and during multiple meetings in England. She made the acquaintance of Blashko, Bülbring, Felberg, Vogt and Vane, at the Oxford meeting of the British Pharmacological Society she met J.H. Gaddum from England, Bovet form Italy, Hopkins from USA, Rothlin from Switzerland, Bacq from Belgium. Her friendship with Gaddum, Vogt and Bülbring lasted for their whole life. Thanks to her stay in Oxford, she had the opportunity to meet during travelling through France the French leading pharmacologists, as Hazard, Cheymol and Lechat. After 1948, with the Soviet power in Czecho-Slovakia, travelling to the West (Western Europe and USA) and many other things started to be extremely difficult. The actual political situation always reflected on science and, of course, affected pharmacology as well. In 1950 she visited the USSR and met there the leading Russian pharmacologists, Anitschkov, Zakusov, Kostojans and Birjukov.

After an informal “pharmacologic” meeting in 1947 in London, at the IUPS congress which took place in Copenhagen (1947), no attention was given to pharmacology. This was why the unified Scandinavian Pharmacological Society organized a post-congress informal pharmacologic meeting with prominent European and American speakers. As she used to say: “*During the next IUPS meeting in Montreal thanks to Meliville, McGill and Fergusson there was the first real world pharmacological meeting”*. C. F. Schmidt, the secretary of the informal pharmacological meeting in Copenhagen wrote to Prof. Rašková to negotiate for establishment of an independent pharmacological body. In the meantime (1955) Prof. Rašková established two independent pharmacological institutes within Charles University, the Department of Pharmacology, Faculty of Pediatrics and the Department of Pharmacology, Faculty of Hygiene (the present 2^nd^ and 3^rd^ Medical Faculties). The highlight came in 1956 during the IUPS congress in Brussels, when the one-day pharmacological meeting after the congress declared itself as a “General Assembly of the International Union of Pharmacology (IUPHAR)”. Consequently, in the IUPS congress in Buenos Aires (1959) Schmidt succeeded in winning approval for the establishment of the “Section of Pharmacology” within the IUPS framework. Prof. Helena Rašková and the Swedish Prof. Börje Uvnäs were two of the European pharmacologists who substantially contributed to the establishment of IUPHAR. In appreciation of these merits, Prof. Rašková was awarded the Gold Medal of the Federation of European Pharmacological Societies (EPHAR).

Due to her efforts, after agreement with the Czechoslovak Physiological Society in 1959, the Czechoslovak Society of Pharmacology was formed. She was repeatedly elected President of this society until 1970. Later she was Honorary President of the Czech Society for Experimental and Clinical Pharmacology and Toxicology and with her sustained vitality she remained in the center of many pharmacological as well as non-pharmacological activities. She used her authority in the foundation of a pharmacological laboratory within the Institute of Organic Chemistry and Biochemistry of the Czechoslovak Academy of Sciences (CzSASci). In 1963, she succeeded to make this laboratory independent and to form the Institute of Pharmacology with headquarters in Prague and with an affiliated branch in Bratislava, together with a farm for laboratory animals in Dobrá Voda, close to Bratislava. The Slovak part of this Institute of Pharmacology of the CzSASci was the basis of the present Institute of Experimental Pharmacology and Toxicology of the Slovak Academy of Sciences.

The network of her closest collaborators (Zdeňk Votava) and first disciples (Jiří Vaněček, Max Wenke, Miroslav Mráz, Vojtěch Grossmann, Václav Trčka, Vojtěch Sobek, Zdena Horáková, Miloš Háva) her scientific children-grandchildren (Ivo Janků, Jiří Elis, Jaroslav Květina, Radan Čapek, Sixstus Hynie, Karel Mašek, Ota Linet, Egon Novák, Ján Štulc, Václav Špičák, Otto Küchel, Vladislav Eybl, Vlado Kovalčík, Jana Machová, Waitzová Dagmar, Pavol Hrdina, Pavel Švec, Jozef Novotný, Otakar Gulda, Milan Šamánek, Miloslav Kršiak, Jan Švihovec, Ladislav Volicer, František Perlík, Radomír Nosál, Zdeněk Zídek, Ondřej Kadlec, Tomáš Sechser, Miroslav Starec, as well as Viktor Bauer the author of this article) were/are holding important posts in pharmacological research not only in the Czech and Slovak Republic but also abroad.

With a specific involvement, she took the initiative and participated in the postgraduate education of pharmacologists from Slovakia and under her auspicies several professors of pharmacology in the Czech and Slovak Republic were established. She used to say: “*One half of my heart belongs to the Czech and the other half to the Slovak pharmacologists*.”

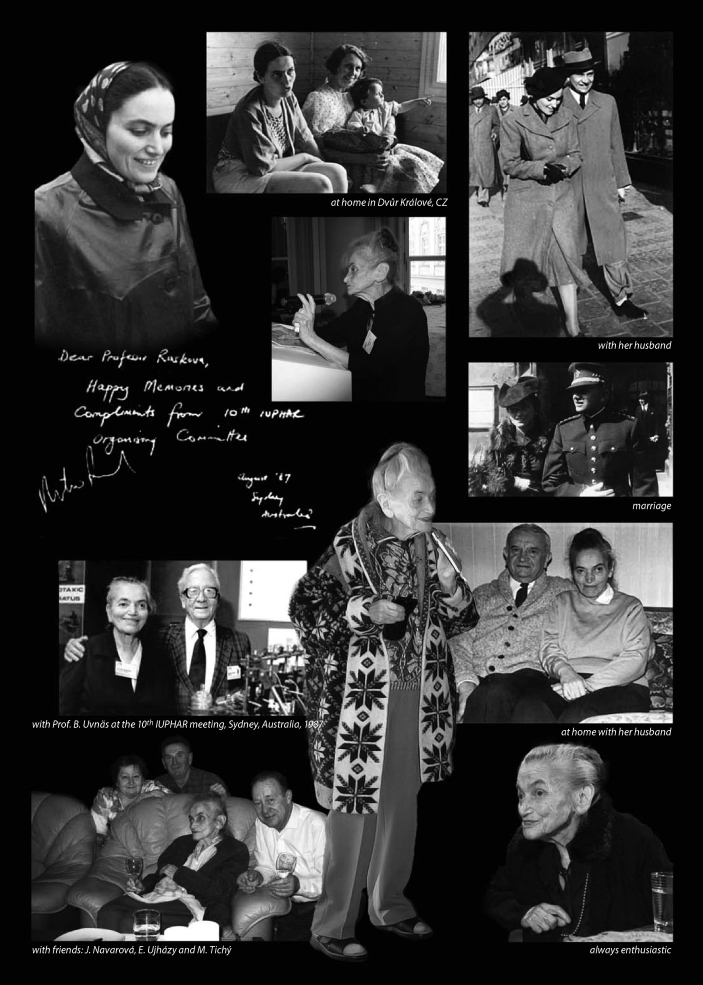



In 1961 the Swedish pharmacologists, in collaboration with some American pharmacologists, organized the first real Pharmacology World Congress in Stockholm. Times were better and Prof. Rašková concentrated all her effort on obtaining from the Czecho-Slovak governmental authorities the necessary written documents to attend the meeting. Although the authorities’ reply was disappointing (“If the next Pharmacology World Congress would be in Prague, visas would be issued”), she succeeded and eventually some people were allowed to attend the World Congress in Stockholm. At present, in the 21^st^ century, it is hard to believe how hard she had to fight and to lobby for these demands. The situation got even worse by the disaster of the construction of the Berlin Wall about two weeks before the Stockholm meeting. She convinced the delegates that if they would vote for Prague, the countries behind the Iron Curtain would have the possibility to communicate with the remaining part of the world. Unbelievably, she succeeded and the second Pharmacology World Congress took part in Prague (1963).

Thanks to her arduous activity and polyglottism (fluent knowledge of Czech, Russian, German, French and English), she managed to maintain communication with world science and leading scientists of both sides (e.g. professors Heymans, Gaddum, Ariens, Burn, Cheymol, Brücke, Bülbring, Trendelenburgh, Eccles, Reuse, Ebashi, Kuriyama on one side and Anitschkov, Zakusov, Kostojans, Birjukov, Kubikovski, Szekeres. Knoll, Jung, *etc.* on the other side). The success of the Prague Congress resulted in, for a number of years in many responsible positions of Prof. Rašková in SEPHAR, eventually IUPHAR, and other organizations. Neither totalitarian practices, restrictions, political barriers nor political pressures orienting Czecho-Slovakia exclusively to the East, were able to eliminate the “cosmopolitan” scientific contacts created by Prof. Rašková for Czecho-Slovak pharmacology. She created possibilities for the young generation to meet leading “western” pharmacologists, and established the possibility for tens of them to stay and have training opportunities in some interesting laboratories in the West and East during the sixties, seventies and eighties.

In addition to the obtained goals in our experimental pharmacology of the 2^nd^ half of the 20^th^ century which are linked with the name of Prof. Helena Rašková, her initiative was of key importance also in our history of “Drug Toxicology” and “Clinical Pharmacology”. In 1963, the Czecho-Slovak toxicological section of the Pharmacological Society was among the founding members of the European Society for the Study of Drug Toxicity (predecessor of the present EUROTOX). Consequently, two of the first European Toxicological Congresses were organized in Prague (1967) and Karlové Vary (1974).

The invasion of Czechoslovakia led by the Soviets on August 21, 1968 changed everything. Her vitality and enthusiasm for science did not abate even in the 1970s and 1980s when the existing political situation forced her to leave the university and the scientific institute which she had helped to establish. For her it meant spending the next 20 years working among cows and calves. She was unable to continue in her research, but was given an opportunity to work “directly in the field” with calf agglomeration. Here again she demonstrated her creativity and reached internationally recognized results with anti-infection conditions and oral rehydration of calves. During this long period of time she used to say: “*If you are old enough and have experience in pharmacology, even such situation can bring some useful results.”*

After splitting Czecho-Slovakia she motivated periodical organizations of Joint Czech and Slovak Pharmacological and Toxicological Meetings, alternately in the Czech and the Slovak Republic.

The recognition of her merits is manifested in dozens of honorary memberships and medals from a number of world scientific societies, university institutes, industry and public organizations.

The range of her scientific activities was voluminous and miscellaneous. She published more then 500 scientific papers and several books dealing with anesthetics, curare-like agents, anti-thyroids, analeptics, hypnotics, antimetabolites, drugs from plants, handbooks of pharmacology, etc. Remarkable international attention was given to her original complex of studies on pharmacology of bacterial toxins, on their effects, on non-specific resistance, as well as on the fate of drugs in the organism.

With her life she fulfilled what Bernard Halpern told her about himself: *“In the first stage I work; in the second stage they build me an institute; and in the third stage I show the institute.”*

All the pharmacologists and toxicologists in the Czech and Slovak Republic remember with special affection Prof. Helena Rašková, our teacher, scientific mother, grandmother and great-grand mother.

*Viktor Bauer*

